# Supplementary material for: Transparent Transfer‐Free Ultrasmall Multilayer Graphene Microelectrodes Enable High Quality Recordings in Brain Slices
Source: Adv Sci (Weinh). 2026 Feb 4;13(20):e17524. doi: 10.1002/advs.202517524 (PMC13067786; doi:10.1002/advs.202517524)
Supplement: Supplementary file 1 — Supporting File: advs73844‐sup‐0001‐SuppMat.pdf. [file ADVS-13-e17524-s001.pdf]

Supporting Information

Transparent Transfer-free Ultrasmall Multilayer Graphene Microelectrodes Enable High Quality Recordings in Brain Slices

Nerea Alvarez de Eulate[1], Christos Pavlou[1], Gonzalo León González[1], María Camarena Pérez[1], Lukas Holzapfel[2], Zhenyu Gao[3], Sten Vollebregt[1], Vasiliki Giagka[1,2]

[1]Department of Microelectronics, Faculty of Electrical Engineering, Mathematics and Computer Science, Delft University of Technology, Delft, The Netherlands.

[2]Department of System Integration and Interconnection Technologies, Fraunhofer Institute for Reliability and Microintegration IZM, Berlin, Germany.

[3]Department of Neuroscience, Erasmus MC, Westzeedijk 353, 3015 AA, Rotterdam, the Netherlands

Device Fabrication

This section provides additional details on the fabrication workflow and system-level integration of the transfer-free multilayer graphene MEAs presented in this work. Figure S1 illustrates the complete MEA–to–Intan acquisition interface, highlighting the mechanical and electrical coupling strategy used for stable multichannel recordings. Figure S2 outlines the wafer-scale fabrication sequence used to produce the transparent multilayer graphene electrodes on fused silica substrates.

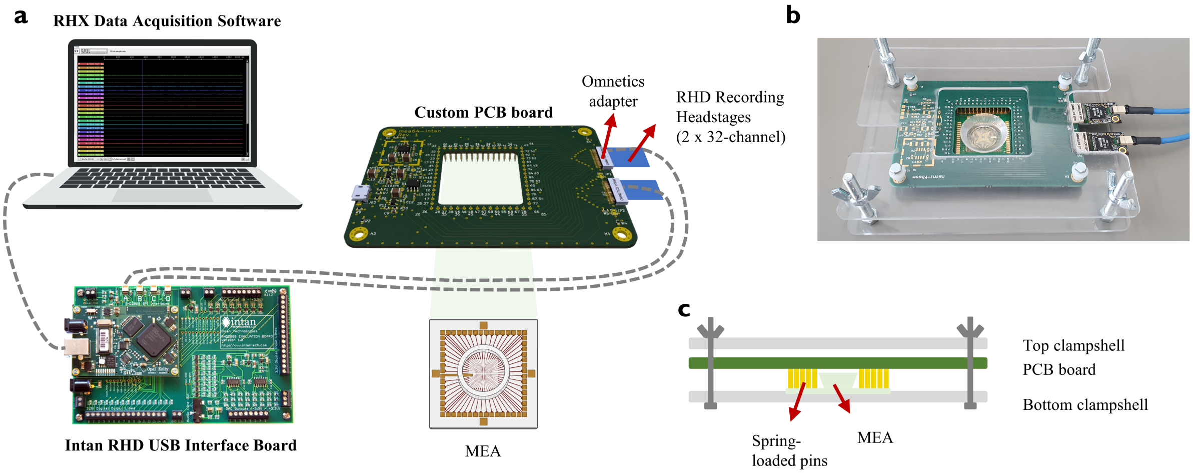

Figure S1. Overview of the MEA acquisition system setup. a. Schematic representation of the connections between the custom PCB board and the Intan recording system. The custom PCB board allows to couple the MEA electrodes, up to a total of 64, to the recording channels from Intan headstages. Spring-loaded pins, soldered to the custom PCB, effectively contact with the MEA pads and route these connections to the Omnetics adapter pads, and in turn to the Intan recording headstage pins. b. Image of the assembled MEA-to-Intan interface. c. Cross-

sectional illustration of the MEA-to-Intan interface. The mechanical parts (top and bottom clampshells) ensure a good contact between the spring-loaded pins from the custom PCB and the MEA pads.

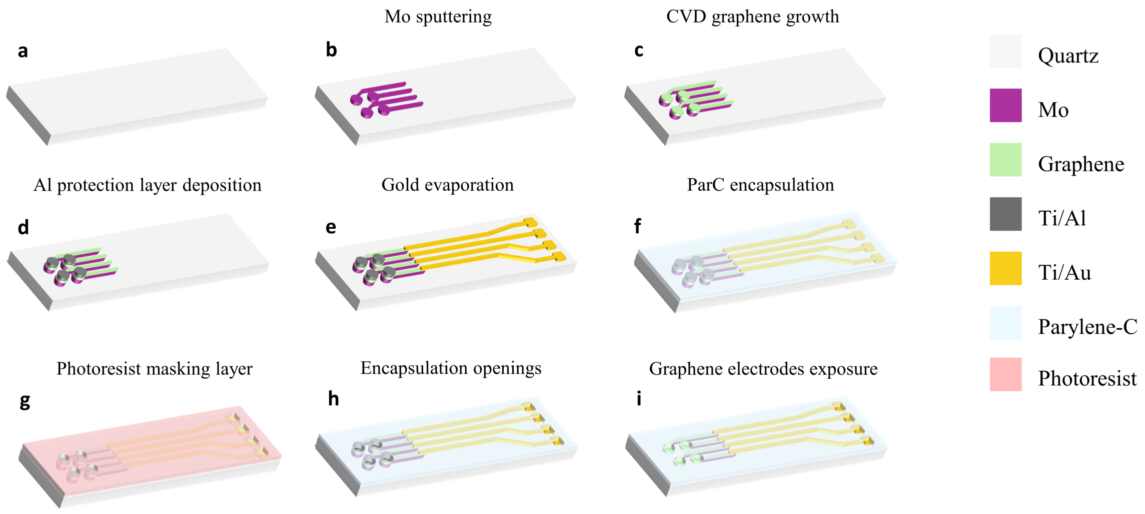

Figure S2. Wafer-scale transfer-free fabrication process steps of graphene MEA electrodes. a. Quartz wafer substrate, b. sputtering of 50 nm Mo layer and patterning, c. CVD multilayer graphene growth, d. Ti/Al deposition and patterning on the microelectrodes, and deposition of a 200 nm Ti layer in the backside (for the later electrostatic clamping in the AMS110 plasma etcher, in step h), e. Ti/Au (10/200 nm) evaporation and lift-off, f. deposition of 1 μm parylene-C encapsulation layer, g. photoresist mask deposition, h. parylene plasma etching, i. Ti/Al and Mo wet etching on the electrode openings.

Multilayer Turbostratic Graphene Characterization

The characterization of the turbostratic graphene grown by CVD on molybdenum presented in this study is summarized in Figure S3 and Figure S4. Raman spectroscopy revealed the multilayer and turbostratic nature of the CVD grown graphene films. Turbostratic graphene refers to a type of multilayer graphene where the layers are rotationally misaligned, leading to weaker interlayer interactions and electronic decoupling.[1] The characteristic G, 2D and D peaks of graphene (~1582, 2660, 1335 cm^{-1}, respectively) were observed, with an I_{2D}/I_G ratio lower than 1, confirming the presence of the multilayer graphene (see Table S1). Additionally, spatially resolved Raman mapping performed on the 10 μm electrodes, see Figure S3b, validated that the single-peak nature of the 2D band is preserved across the exposed recording area. The maps of 2D peak position and full width at half maximum, together with the corresponding histograms of 2D peak position (ω_{2D}), FWHM (Γ_{2D}) and I_{2D}/I_G ratios (Figure S3), show a single-Lorentzian 2D line shape at every spectrum. Across the mapped area, ω_{2D} is distributed between 2659 and 2669 cm^{-1} (centered around ≈ 2665 cm^{-1}), Γ_{2D} mainly in the 65–75 cm^{-1} range, and $I_{2D}/I_G \approx 0.6 \pm 0.1$. These parameters vary smoothly across the electrode without domain-like changes or 2D-peak splitting, which strongly suggested that the graphene layers were turbostratic, as they did not exhibit the complex splitting of more ordered stacking.[1] This interpretation is further supported by a previous work illustrating independent TEM analysis of Mo-grown multilayer graphene produced using the same catalyst system, which shows an enlarged interlayer spacing (> 0.34 nm), a structural hallmark of turbostratic multilayer graphene.[2] The I_D/I_G ratio, indicative of the amount of defects, was low, $I_D/I_G = 0.35$ (see Table S1), right after graphene growth on Mo catalyst layer on a quartz substrate. The

number of defects slightly increased as the graphene layer was post-processed. Additional Raman spectra at different stages of the fabrication process are provided in Figure S4.

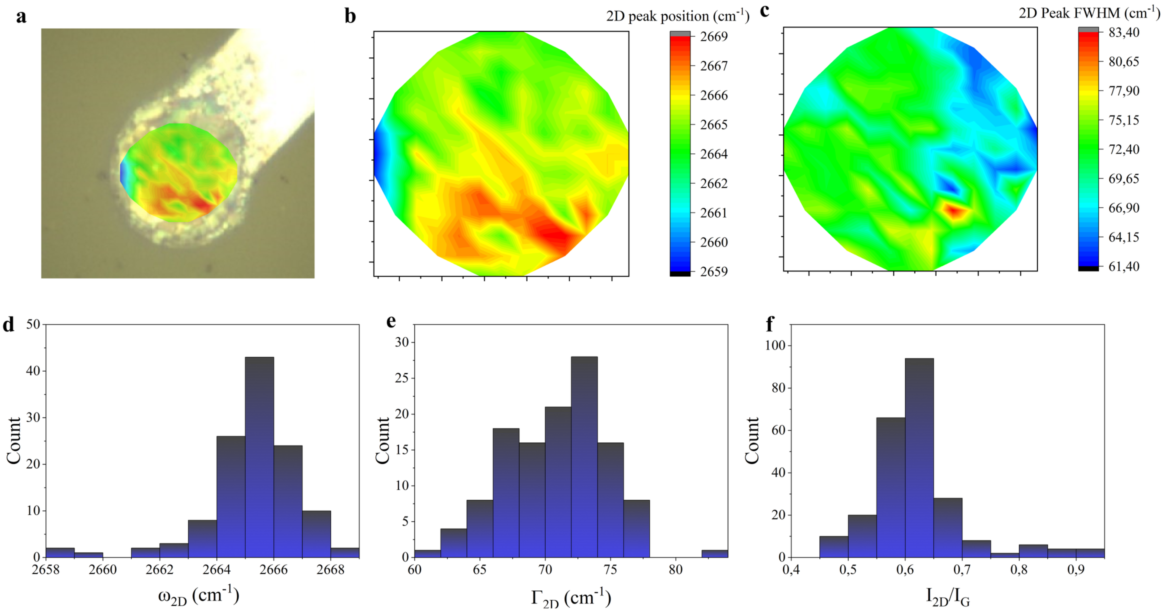

Figure S3. Raman mapping analysis of multilayer turbostratic graphene obtained from the fabricated 10 μm microelectrodes. a. Optical micrograph of a 10 μm recording site with the Raman map overlaid. b. Contour map of the 2D peak position (ω_{2D}) obtained from single-Lorentzian fits at each pixel. c. Corresponding map of the 2D peak full width at half maximum (Γ_{2D}). Maps in (b,c) were acquired with a 633 nm excitation laser over a 10 μm diameter circular window centered on the electrode, using a lateral step of 1 μm (121 spectra in total). d. Histogram of ω_{2D} showing a narrow distribution around ~2665 cm^{-1}. e. Histogram of Γ_{2D} with values mainly in the 65–75 cm^{-1} range. f. Histogram of the intensity ratio I_{2D}/I_G.

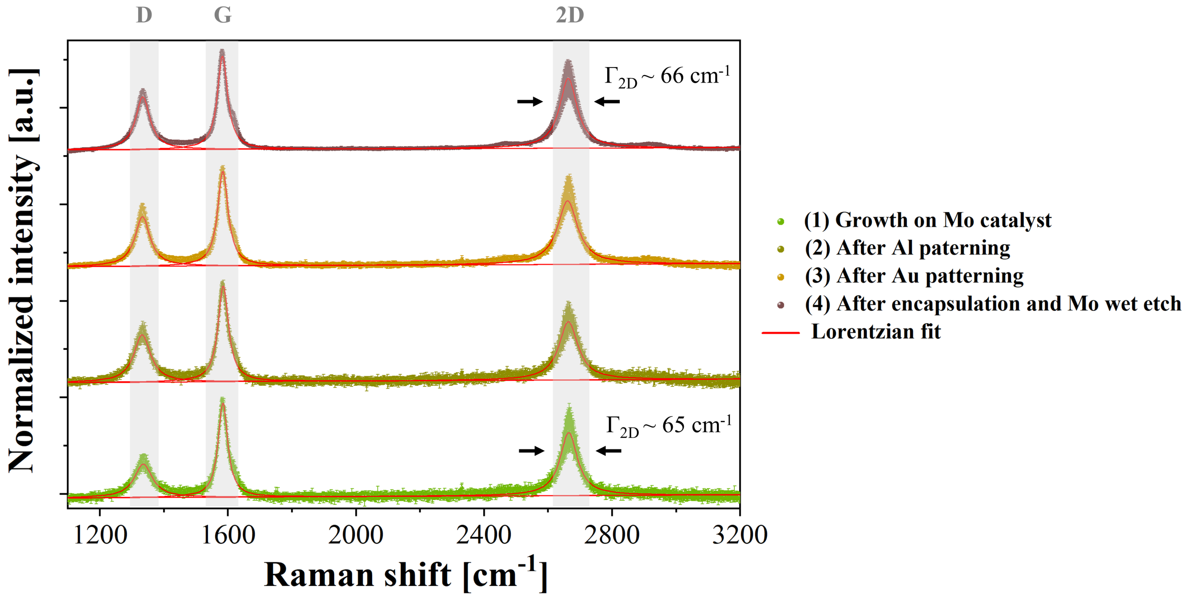

Figure S4. Raman spectra of multilayer turbostratic graphene obtained at different stages of the microfabrication process: (1) Right after graphene grown on Mo catalyst on a quartz substrate, (2) After Al sacrificial protective layer deposition and patterning, (3) after Au tracks deposition and patterning, (4) after parylene C encapsulation, openings formation and Mo wet etching. Each graph line shows the average and standard deviation of 5-point measurements. The G and 2D bands retain their characteristic positions and single-Lorentzian 2D line shape, with $\Gamma_{2D} \approx$ 65–66 cm^{-1}, confirming the preservation of the turbostratic structure throughout processing.

Process Stage	I_D/I_G ratio	I_{2D}/I_G ratio
Growth on Mo catalyst	0.35 ± 0.09	0.72 ± 0.25
After Al patterning	0.49 ± 0.11	0.64 ± 0.21
After Au patterning	0.55 ± 0.14	0.78 ± 0.19
After encapsulation and Mo etch	0.50 ± 0.08	0.68 ± 0.10

Table S1. Raman ratios of transfer-free multilayer graphene obtained from the spectra at different stages of the process.

Electrodes Characterization: Electrochemical Impedance Spectroscopy

	**Impedance magnitude at 1 kHz (kΩ)**			
**Electrode diameter (µm)**	10	30	50	100
Multilayer graphene	5680 ± 1370	550.2 ± 246.6	263.6 ± 44.0	31.1 ± 12.2

Table S2. Impedance magnitude at 1 kHz (kΩ) of transfer-free multilayer graphene electrodes of various sizes, ranging from 10 to 100 µm in diameter. The average and standard deviation for electrode sizes of 30, 50 and 100 µm are calculated from 10 measurements, and for 10 µm electrodes from the average of 42 measurements.

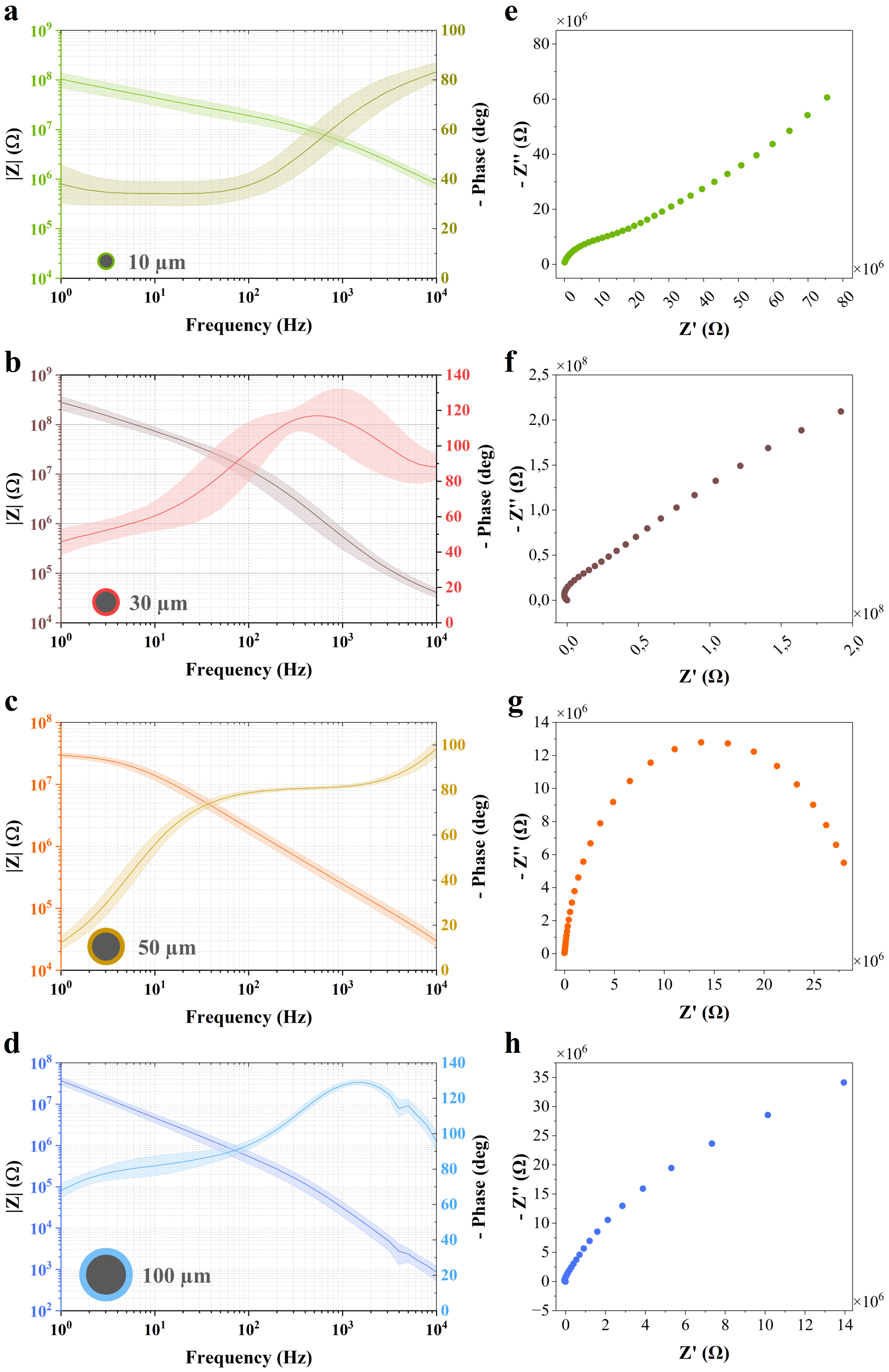

Figure S5. Electrochemical impedance spectroscopy (EIS) characterization of fabricated transfer-free multilayer graphene electrodes. (a–d) Averaged impedance spectra, including magnitude and phase, of microelectrodes with diameters of (a) 10 μm ($n = 42$), (b) 30 μm ($n = 10$), (c) 50 μm ($n = 10$), and (d) 100 μm ($n = 10$). Shaded regions show standard deviation. (e–h) Corresponding average Nyquist plots for the same electrodes with diameters of (e) 10 μm, (f) 30 μm, (g) 50 μm, and (h) 100 μm.

Benchmarking of transparent neural recording interfaces. Electrode impedance and SNR values

Table S3. Transparent neural recording arrays. Comparison of previously reported transparent recording electrodes with the transfer-free graphene electrodes presented in this work.

Electrode material	Electrode surface area (µm²)	Impedance at 1 kHz (kΩ)	Area-normalized impedance (Ω·cm²)	Transparency (%)	SNR (dB) [reported][a)]	Recording type	Ref.
Monolayer graphene doped with acid nitric	2500 (50 × 50)	541	13.5	90	32.2 dB [40.8]	In vivo Drug-induced epileptiform activity in cortical surface	[3]
Four-layer graphene	31416 (D = 200)	243.5 ± 5.9	76.50 ± 1.85	90	–	In vivo Electrical-evoked potentials at somatosensory cortex	[4]
Monolayer graphene	314 (D = 20)[b)]	3500 ± 500	10.99 ± 1.57	–	24.1 dB [16]	In vitro Spontaneous activity from rat embryonic cortical neurons	[5]
Two-layer graphene doped with acid nitric	2500 (50 × 50)	908	22.7	90	> 14 dB [> 5]	In vivo Drug-induced epileptiform activity in cortical surface	[6]
Interlayer-doped double-layer graphene with Pt NPs	314 (D = 20)	250 ± 56	0.78 ± 0.18	90	–	In vivo Stimulus-evoked potentials at visual cortex	[7]
Monolayer graphene coated with PEDOT:PSS	707 (D = 30)	166 ± 13	1.17 ± 0.09	84	–	In vitro Spontaneous activity from cardiac cell culture	[8]
PEDOT:PSS	707 (D = 30)	56 ± 8	0.39	75	17.7 dB [7.7]	In vitro Spontaneous activity from primary neuronal cell culture	[9]
Graphene – ITO	314 (D = 20)	1450	4.55	80	24.1 dB [16]	In vivo Spontaneous activity in the hippocampus of freely moving mice	[10]
Transfer-free multilayer graphene	79 (D = 10)	5680 ± 1370	4.46 ± 1.08	50	18.6 dB [8.5]	Ex vivo Spontaneous activity from cerebellar brain slice	This work

[a)] The method for calculating the SNR varies across studies, as there is no standardized approach for its computation. The values enclosed in brackets correspond to the original SNR values, on a linear scale, as reported in the study. [b)] Kireev et al.[5] , in fact, fabricated graphene electrodes as small as 10 µm in diameter, but did not record neural activity.

Supplementary References

[1] D. R. Lenski, and M. S. Fuhrer, “Raman and optical characterization of multilayer turbostratic graphene grown via chemical vapor deposition,” *Journal of Applied Physics* **110**, no. 1 (2011).

[2] L. N. Sacco, A. Dobrowolski, B. Boshuizen, et al., “Controlling the number of layers of Mo-grown CVD graphene through the catalyst thickness,” *Diamond and Related Materials* **154** (2025), 112195.

[3] D. Kuzum, H. Takano, E. Shim, et al., “Transparent and flexible low noise graphene electrodes for simultaneous electrophysiology and neuroimaging,” *Nature Communications* **5**, 5259 (2014).

[4] DW. Park, A. Schendel, S. Mikael, et al., “Graphene-based carbon-layered electrode array technology for neural imaging and optogenetic applications,” *Nature Communications* **5**, 5258 (2014).

[5] D. Kireev, S. Seyock, J. Lewen, V. Maybeck, B. Wolfrum, and A. Offenhäusser, “Graphene Multielectrode Arrays as a Versatile Tool for Extracellular Measurements,” *Advanced Healthcare Materials* **6**, no. 12 (2017), 1601433.

[6] N. Driscoll, R.E. Rosch, B.B. Murphy, et al., “Multimodal in vivo recording using transparent graphene microelectrodes illuminates spatiotemporal seizure dynamics at the microscale,” *Communications Biology* **4**, 136 (2021).

[7] M. Ramezani, JH. Kim, X. Liu, et al., “High-density transparent graphene arrays for predicting cellular calcium activity at depth from surface potential recordings.” *Nature Nanotechnology* **19** (2024), 504–513.

[8] P. Kshirsagar, S. Dickreuter, M. Mierzejewski, et al., “Transparent Graphene/PEDOT:PSS Microelectrodes for Electro- and Optophysiology,” *Advanced Materials Technologies* **4**, no. 1 (2019), 1800318.

[9] S. Middya, V.F. Curto, A. Fernández-Villegas, et al., “Microelectrode Arrays for Simultaneous Electrophysiology and Advanced Optical Microscopy,” *Advanced Science* **8**, no. 13 (2021), 2004434.

[10] M. Yuan, F. Li, F. Xue, et al., “Transparent, flexible graphene–ITO-based neural microelectrodes for simultaneous electrophysiology recording and calcium imaging of intracortical neural activity in freely moving mice,” *Microsystems & Nanoengineering* **11**, no. 32 (2025).
